# Surgeon views regarding the adoption of a novel surgical innovation into clinical practice: systematic review

**DOI:** 10.1093/bjsopen/zrad141

**Published:** 2024-01-24

**Authors:** Nagarjun N Konda, Thomas L Lewis, Hugh N Furness, George W Miller, Andrew J Metcalfe, David R Ellard

**Affiliations:** Warwick Clinical Trials Unit, Warwick Medical School, The University of Warwick, Coventry, UK; Department of Trauma and Orthopaedic Surgery, University Hospitals Coventry & Warwickshire, Coventry, UK; Department of Trauma and Orthopaedic Surgery, King’s College Hospital NHS Foundation Trust, London, UK; Department of Trauma and Orthopaedic Surgery, Imperial College London, London, UK; Department of Trauma and Orthopaedic Surgery, Bart’s and the London NHS Foundation Trust, London, UK; Warwick Clinical Trials Unit, Warwick Medical School, The University of Warwick, Coventry, UK; Department of Trauma and Orthopaedic Surgery, University Hospitals Coventry & Warwickshire, Coventry, UK; Warwick Clinical Trials Unit, Warwick Medical School, The University of Warwick, Coventry, UK; Department of Trauma and Orthopaedic Surgery, University Hospitals Coventry & Warwickshire, Coventry, UK

## Abstract

**Background:**

The haphazard adoption of new surgical technologies into practice has the potential to cause patient harm and there are many misconceptions in the decision-making behind the adoption of new innovations. The aim of this study was to synthesize factors affecting a surgeon’s decision to adopt a novel surgical innovation into clinical practice.

**Methods:**

A systematic literature search was performed to obtain all studies where surgeon views on the adoption of a novel surgical innovation into clinical practice have been collected. The databases screened were MEDLINE, Embase, Science Direct, Scopus, the Web of Science, and the Cochrane Library of Systematic Reviews (last accessed October 2022). Innovations covered multiple specialties, including cardiac, general, urology, and orthopaedics. The quality of the papers was assessed using a 10-question Critical Appraisal Skills Programme (CASP) tool for qualitative research.

**Results:**

A total of 26 studies (including 1112 participants, of which 694 were surgeons) from nine countries satisfied the inclusion and exclusion criteria. Types of study included semi-structured interviews and focus groups, for example. Themes and sub-themes that emerged after a thematic synthesis were categorized using five causal factors (structural, organizational, patient-level, provider-level, and innovation-based). These themes were further split into facilitators and barriers. Key facilitators to adoption of an innovation include improved clinical outcomes, cost-effectiveness, and support from internal and external stakeholders. Barriers to adoption include lack of organizational support and views of senior surgeons.

**Conclusion:**

There are multiple complex factors that dynamically interact, affecting the adoption of a novel surgical innovation into clinical practice. There is a need to further investigate surgeon and other stakeholder views regarding the strength of clinical evidence required to support the widespread adoption of a surgical innovation into clinical practice.

## Teaser

Factors affecting the adoption of surgical innovations into healthcare are not well understood. The aim of this systematic review was to identify surgeons’ views regarding the factors that affect the adoption of effective novel surgical innovations into clinical practice. This may facilitate increased adoption of surgical practice with improved health provision for patients.

## Introduction

The introduction of novel surgical innovations into clinical practice has helped contribute to significant improvements in the duration and quality of patients’ lives^1^. Innovation in surgery has led to new instruments, equipment, and operative procedures that contribute to reduced morbidity and mortality^2,3^. Examples include robotic procedures and the integration of three-dimensional printing for operative planning. Surgeons often adopt new technologies into practice, despite poor evidence regarding the efficacy of an innovation^4^. The haphazard adoption of innovative surgical technologies without proper evaluation has the potential to cause significant harm to patients^1^.

Previous discredited innovative technologies that have caused harm include transvaginal mesh, which resulted in a high number of litigation cases due to mesh erosion, infection, and dyspareunia, and metal-on-metal hip prostheses^5,6^. The idea, development, exploration, assessment, long-term study (IDEAL) framework provides a set of recommendations for how evaluation should be conducted at each stage of the surgical innovation process^1,7^. Previous research has shown uptake of the IDEAL framework to be slow or followed incorrectly, and adoption of surgical innovations still happens on an ad hoc basis, with no barriers to a unit adopting a new or untested technique based on scientific evidence^4,8^.

Chaudoir *et al*.^9^ identified five causal factors (structural, organizational, patient-level, provider-level, and innovation-based) that predict the diffusion and sustainability of health innovations. It has been shown that the interplay and dynamic relationship between individual factors will lead to variation in the rate of adoption of an innovation into clinical practice. Innovations that are more complex are less likely to be successfully adopted, scaled up, and sustained. This has led to alternative framework proposals to help predict and evaluate the success of a technology-supported health or social care programme, although there is limited evidence regarding the adoption of surgical innovations^10^.

The aim of this systematic review was to explore and synthesize the qualitative evidence concerning surgeon views regarding the adoption of a novel surgical innovation (specifically chosen due to the role of surgeons in the decision to adopt a novel surgical innovation that has been highlighted in the literature). It will provide deeper insights into the adoption, implementation, and diffusion of surgical innovations. Such information may guide further research and may also identify areas where better systems, policies, or education could improve the safe introduction of new procedures and technologies into practice.

## Methods

The full details of the methods can be found in the protocol paper^11^, which was prospectively written in line with PRISMA-P guidelines^12^. A systematic review of qualitative research was performed following PRISMA guidelines^13^. The systematic review was prospectively registered on PROSPERO, the international prospective register of systematic reviews (CRD42017076715)^14^. Reporting of this review was guided by the Enhancing Transparency in Reporting the Synthesis of Qualitative Research (ENTREQ) framework^15^.

### Literature search

**Fig. 1:**
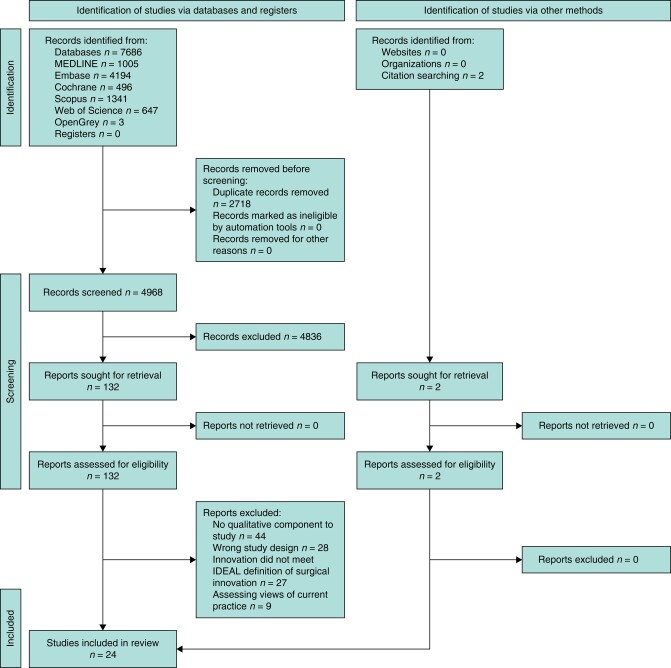
PRISMA flow diagram IDEAL, idea, development, exploration, assessment, long-term study.

A systematic search of six electronic databases was conducted (MEDLINE, Embase, Scopus, the Web of Science, OpenGrey and the Cochrane Library of Systematic Reviews). Searches were conducted from inception until September 2017 and updated in October 2022. The full detailed search strategy is available in *Table S1*. *Figure 1* is a PRISMA diagram demonstrating the study selection and data extraction process. Initially, titles and abstracts were screened independently by at least two reviewers (N.N.K. and T.L.L.). Any disagreement was referred to a third reviewer. Screening and extraction of data records was managed using Covidence—a web-based systematic review manager^16^. Ineligible articles were removed and the full text of all potentially relevant articles was retrieved and reviewed for eligibility by two independent authors (N.N.K. and T.L.L.).

### Study selection

Studies that included qualitative methods (for example, interviews, focus groups, and open-ended questionnaires) were included. For the purposes of this review, the definition of surgical innovation utilized by the IDEAL framework was used, which describes an innovative procedure in surgery as ‘a new or modified surgical procedure that differs from currently accepted local practice, the outcomes of which have not been described, and which may entail risk to the patient’^17^.

### Inclusion criteria

Studies were only included if they met the following inclusion criteria: a report on the adoption of a novel innovation into clinical practice; the innovation described met the IDEAL framework definition of a surgical innovation^17^; included the views of at least one surgeon in the qualitative analysis; the innovation was considered novel at the time of investigation; and English language only.

The exclusion criteria included: studies that were quantitative in nature; and studies with closed-ended questionnaires. Studies related to innovations in the field of dentistry and data published in abstract form only were specifically excluded.

### Quality assessment

The quality of the papers was assessed using a 10-question Critical Appraisal Skills Programme (CASP) tool^18^ for qualitative research^19,20^. Studies were grouped into low (0–3 points), medium (4–7 points), and high (8–10 points) quality. Low-quality studies were not excluded, but caution was taken when interpreting their results.

### Data extraction and analysis

This qualitative systematic review utilized a thematic synthesis (a type of narrative synthesis)^21,22^. Qualitative data from mixed-method studies were screened for inclusion and included if the qualitative component was relevant and reported separately. Three authors (N.K.K., T.L.L., and H.N.F.) independently examined each line of each study’s findings to create codes that described meaning and content. Findings were sought throughout each study, not just from the Results sections.

## Results

Adoption of surgical innovations was often described in terms of facilitators and barriers, so study findings are broadly categorized under these headings. The facilitators were captured into 22 sub-themes and the barriers into 17 sub-themes. These themes were similar to those found by previous reviews^9,23^. Themes and sub-themes were then categorized using the five causal factors described by Chaudoir *et al*.^9^ that affect the implementation of innovation in healthcare (structural, organizational, patient-level, provider-level, and innovation-based).

Italic text within quotation marks identifies primary data quotes from participants. The confidence in each review finding was assessed using the GRADE-CERQual framework^24^.

A total of 26 studies (including 1112 participants, of which 694 were surgeons) met the inclusion criteria^25–50^ (*Fig. 1*). The characteristics of these studies and their participants can be seen in *Table 1*. The studies investigated the adoption of a range of surgical innovations and used a range of focus groups, in-depth interviews, semi-structured interviews, face-to-face interviews, workshops, open-ended surveys, and telephone interviews. Studies were conducted in nine different countries (the USA (11), Canada (5), the UK (4), Germany (1), Australia (1), Belgium and the Netherlands (2), an unnamed country in West Africa (1), and Norway (1)). The study settings included community hospitals and academic hospitals, and some studies involved surgeons who were members of various societies.

**Table 1:** Characteristics of the included studies

Study	Study type	Surgical innovation	Specialty	Method of analysis	Study population	Study settings	Study aims	Study quality
Abrishami 2014 Netherlands	Semi-structured group interviews	da Vinci surgical robot	Urology	Contextualization	Urologists (8), patients with prostate cancer (4), hospital managers (3), private health insurance companies (3), healthcare journalists (3), national policymakers (2), hospitals’ technical assistants (2), an organizer of international medical congresses (1), an operation theatre nurse (1), and a clinical epidemiologist (1)	Dutch healthcare system	To gain an understanding of the adoption dynamics of healthcare innovations by examining one specific case, namely the da Vinci robot in the Netherlands	Medium
Acharya 2009 USA	Mixed methods (qualitative survey)	Minimally invasive surgery	Urology	Descriptive analysis	Urological surgeons (85)	Members of the Society of Urologic Oncology	To clarify whether open surgeons have modified their techniques, surgical equipment, and perioperative management using minimally invasive surgery to treat urological malignancies	Low
Altschuler 2021 USA	Semi-structured one-on-one interviews	Percutaneous Tuohy needle (laparoscopic) technique for paediatric inguinal hernia repair	Paediatric surgery	Thematic analysis	Paediatric surgeons (6) and paediatric urologists (3)	Community-based healthcare system	To identify attitudes and practices regarding the adoption of surgical innovations in paediatric surgeons and paediatric urologists	Medium
Beech 1992 UK	Mixed methods (postal survey with qualitative component)	Day surgery	General surgery	Descriptive analysis	Consultant surgeons (240) from multiple surgical specialties	Consultant surgeons covering 53 districts and 80 hospitals across the National Health Service	To examine the managerial and clinical incentives for day surgery in the UK as a means of assessing the reasons for its low rate of adoption	Medium
Brattheim 2010 Norway	Semi-structured interviews	Endovascular aortic aneurysm repair	Vascular surgery	Thematic analysis	Vascular surgeons (5) and interventional radiologists (7)	One university hospital and two local hospitals in Norway	To explore the social and material context of expertise development and refinement of a novel practice while knowledge and expertise are progressing	High
Choy 2013 West Africa	Semi-structured one-on-one interviews	Laparoscopic surgery	General surgery	Combination of methodologialc, data, and researcher triangulation	Surgeons (9), nurses (3), an anaesthetist (1), and a pharmacist (1)	Single large hospital that is both a regional general hospital and a national referral hospital	To explore and analyse the potential barriers to the adoption of laparoscopic surgery with a view to inform the future development of a laparoscopic surgical training programme in lower-middle-income countries	Medium
Collins 2015 USA	Semi-structured face-to-face interviews	ACS	General surgery	Inductive approach and thematic analysis	Eighteen surgeons (including current section/division chiefs for trauma surgery and/or EGS, two department chairs, and two senior surgeons)	Eighteen teaching hospitals in the USA	To understand the benefits and drawbacks of ACS and to determine what senior surgeons see for the future of this new specialty	Medium
Danjoux 2007 Canada	Semi-structured one-on-one interviews	Endovascular aortic aneurysm repair	Vascular surgery	Modified thematic analysis	Vascular surgeons (3), a radiologist (1), and a hospital decision-maker (1)	Single large, urban university academic health sciences centre in Toronto, Canada	To describe and evaluate the adoption of a new health technology used by surgeons for the treatment of aortic aneurysms called endovascular aneurysm repair (EVAR)	High
Davey 2011 UK	Mixed methods (semi-structured interviews)	Computer-assisted surgery, hip and knee arthroplasty	Trauma and orthopaedic surgery	Thematic analysis	Twenty-seven trauma and orthopaedic surgeons	Twenty-eight different centres across the UK	To provide an overview of the new technologies involved in orthopaedic surgery at the time of the study (2006–2007) and report on the results of a questionnaire that recorded the opinions of surgeons regarding the introduction of new technologies, with specific relevance to computer-assisted surgery	Low
Dharampal 2016 Canada	Semi-structured interviews	SSC	Multiple surgical specialties	Grounded theory, inductive thematic analysis	Surgeons (12), anaesthetists (10), and operating room nurses (9)	Three acute care hospitals in Calgary, Canada	To determine the attitudes of healthcare providers toward the SSC that may impact its adoption and compliance in Calgary, Canada	High
Edmondson 2001 USA	Semi-structured one-on-one interviews	Minimally invasive cardiac surgery	Cardiothoracic surgery	Thematic analysis	One hundred and sixty-five interviews in total, including one to three people in each of the four operating theatre team roles (surgeons, anaesthetists, nurses, and perfusionists)	Sixteen varied hospitals in the USA	To explore the implementation process and propose a process model for establishing new routines when adopting innovative technology	Medium
Gold 2014 USA	Individual interviews	Accelerated partial breast radiotherapy	Breast surgery	Thematic framework approach	Breast surgeons (17), and radiation oncologists (19)	Mixture of academic and non-academic settings across the USA (number not specified)	To explore the decision-making experience of physicians who had to decide whether to adopt this technology	High
Hinoul 2010 Belgium and Netherlands	Mixed methods (consensus)	Needle suspension techniques with mesh to treat urogenital prolapse	Urogynaecology	Mixed model analysis	Twenty opinion leaders (defined as academic pelvic floor surgeons, active members in urogynaecological societies, and/or high-volume surgeons)	Members of the Flemish and Dutch Societies for Obstetrics and Gynaecology	To determine which characteristics of proposed innovative surgical procedures influence the choice of pelvic floor surgeons when considering the use of mesh prostheses in the surgical treatment of urogenital prolapse	Low
Lambert-Kerzner 2018 USA	Focus groups and interviews	SURPAS	Multiple surgical specialties	Matrix and reflexive analysis	Surgeons (21), intensive care (1), anaesthetists (2), a biostatistician (1), medical students (2), administrators (20), and patients (24)	Academic tertiary referral centre in Colorado	The evaluation of SURPAS to optimize its development and implementation using qualitative methodology with focus groups and individual interviews of patients, surgical providers, and administrators	Medium
Lang 2005 USA	Semi-structured one-on-one interviews	Carotid angioplasty and stenting	Vascular surgery	Intuitive and thematic analysis	Fifteen vascular surgeons	Five medical centres across the USA	Exploration of physicians’ views about the safety and efficacy of carotid angioplasty and stenting, and the negotiation of professional boundaries in the treatment of carotid stenosis	Medium
Leggott 2015 USA	Mixed methods (semi-structured one-on-one interviews)	Transformation of anaesthesia for ambulatory orthopaedic surgery	Trauma and orthopaedic surgery	Inductive iterative thematic analysis	Orthopaedic surgeons (8), anaesthetists (4), and a nurse administrator (1)	Single large hospital in the USA	To identify key factors in the decision-making process in the implementation and acceptance of the innovation	Medium
Luxford 2006 Australia	Workshop	Best-practice guidelines using a matrix tool	General surgery	Thematic analysis	Colorectal surgeons (24), oncologists (16), and others (10; general surgeons, gastroenterologists, policy makers, and psychologists)	The Colorectal Group of the Victorian Cooperative Oncology Group	To pilot a matrix tool and assess its usefulness for individuals and organizations aiming to develop strategies to promote guideline implementation in cancer care	Low
Marcus 2014 UK	Mixed methods (qualitative survey)	Endoscopic and assisted neurosurgical approaches	Neurosurgery	Descriptive analysis	Consultant surgeons (40)	Members of the Society of British Neurosurgeons	To assess the technical challenges of neuroendoscopy, and the scope for technological innovations to overcome these barriers	Low
Merkel 2015 Germany	Problem-centred interviews	TAVI	Cardiothoracic surgery/cardiology	Content and thematic analysis	Cardiologists (9) , and a cardiothoracic surgeon (1)	German university hospitals	To identify and analyse factors affecting the implementation and diffusion of the procedure in hospitals using a qualitative application of the diffusion of innovations theory	High
Powers 2021 USA	Semi-structured one-on-one interviews	Multiple innovations in otolaryngology	Otolaryngology	Thematic analysis	Otolaryngologists (19)	Tertiary academic centre in the USA	To identify barriers and facilitators to adoption of a new surgical procedure via an implementation science framework to characterize associated socio-emotional, clinical, and decision-making processes	Medium
Russ 2015 UK	Semi-structured one-on-one or telephone interviews	WHO SSC	Multiple surgical specialties	Inductive approach followed by thematic analysis	Surgeons (37), anaesthetists (31), nurses (23), ODPs (18), and radiographers (10)	Ten varied hospitals in the UK	To evaluate how the WHO SSC was implemented across hospitals in England; to identify barriers and facilitators toward implementation; and to draw out lessons for implementing improvement initiatives in surgery/healthcare more generally	High
Santry 2014 USA	Semi-structured one-on-one interviews	ACS	General surgery	Investigator triangulation using an inductive approach to develop a final taxonomy of codes organized by themes	Eighteen surgeons (including current section/division chiefs for trauma surgery and/or EGS, two department chairs, and two senior surgeons)	Eighteen teaching hospitals in the USA	To understand how ACS is currently implemented in the USA across hospitals in varied geographical locations and practice settings	High
Sharma 2006 Canada	Semi-structured interviews	Advanced laparoscopic surgery	General surgery	Modified thematic analysis	General surgeons (3), a medical programme director (1), a nurse educator (1), and an operating room manager (1)	One community hospital in Toronto, Canada	Exploration of the current decision-making processes for the adoption of advanced laparoscopic surgery	High
Stafinski 2010 Canada	Workshop	Thirteen separate technologies	Multiple surgical specialties	Content and relational analysis	Eighteen surgeons from several technology- intensive clinical specialties (including cardiac surgery, neurosurgery, and orthopaedic surgery)	Workshop in Alberta, Canada	To pilot an approach to engage surgeons in identifying emerging technologies for health technology assessment	Medium
Vanderveen 2007 USA	Semi-structured one-on-one interviews	SLNB	Breast surgery	Network and thematic analysis	General surgeons (27) and surgical oncologists (11)	A single metropolitan area	To identify decision- making factors underlying SLNB implementation and learning patterns in one community, and to determine whether personal and practice characteristics affected time of adoption and learning sources	High
Wright 2011 Canada	Semi-structured one-on-one interviews	SLNB	Breast surgery	Grounded theory analysis and inductive approach	Surgeons (21), pathologists (5), nuclear medicine physicians (7), and administrators (10)	Range of representatives from Cancer Care Ontario	To explore individual, institutional, and policy factors that may have influenced SLNB adoption	High

ACS, acute care surgery; EGS, emergency general surgery; SSC, surgical safety checklist; SURPAS, surgical risk preoperative assessment system; TAVI, transcather aortic valve implementation; ODPs, operating department practitioners; SLNB, sentinel lymph node biopsy.

### Quality assessment

The majority of included studies were judged as medium or high quality, as shown in *Table 1*. In many studies, consideration of the potential impact of the relationship between the researcher and the participants on findings was not adequately considered and it was often unclear whether ethical issues had been taken into consideration. Further detail regarding risk-of-bias analysis can be found in *Fig. S1*.

### Thematic synthesis of findings

The main findings of the review, shown in *Table 2*, showed that there were 22 themes that facilitated the adoption of a surgical innovation. These were categorized according to the five causal factors described by Chaudoir *et al*.^9^, as described above. Specific barriers to the adoption of a surgical innovation were also categorized according to the causal factors described by Chaudoir *et al*.^9^ (*Table 3*).

**Table 2:** Themes and sub-themes that facilitate adoption of a surgical innovation into clinical practice

Causal factor	Key points	References	Confidence in the evidence	Explanation of confidence finding
**Innovation-based**				
Clinical outcomes and efficiency	Better innovation-specific clinical outcomes (for example, resection margins, post-operative complication rate, and blood loss) Reduced morbidity/mortality Reduced duration of hospital stay Reduced time to return to work Enable more patients to undergo treatment (for example, high risk patients) Improved theatre efficiency (for example, length of cases and time to set up equipment)	^25,26,28,29,30,31,32,33,35,36,37,38,39,41,42,43,44,45,46,47,48,50^	High	Twenty-two studies with minor to significant methodological limitations. Highly relevant with high coherence. High adequacy.
Cost-effectiveness	Initial costs and ongoing costs associated with innovation Reimbursement costs	^25,26,27,28,32,35,36,37,41,43,44,45^	High	Twelve studies with minor to significant methodological limitations. Highly relevant with moderate coherence. High adequacy.
Education and training	Formal surgical training programme Use of simulation and other preparation Use of credentialing to ensure competence Increased training opportunities for surgeons	^26,28,30,34,36,37,40,42,43,44,46,47^	High	Twelve studies with minor to significant methodological limitations. Highly relevant with moderate coherence. High adequacy.
Innovation-specific adoption properties	Ease of adoption into existing clinical practice and pathways Adapting innovation to fit specific clinical circumstances Compatibility and complexity of innovation	^33,34,42,45,47,48,49,50^	High	Eight studies with minor to moderate methodological limitations. Highly relevant with moderate coherence and adequacy.
Ownership of innovation	Which specialty ‘owns’ the innovation Negotiation of specialty boundaries Encouraging staff buy-in to own the innovation	^30,32,33,35,37,41,42,47^	High	Eight studies with minor methodological limitations. Highly relevant with high coherence. Moderate adequacy.
Scientific evidence	National/local conferences Opinion leaders Peer-reviewed publications Surgeon belief about benefits of innovation Generalizing evidence from academic to community settings	^25,28,32,35,36,37,41,45,46,49^	High	Ten studies with minor to significant methodological limitations. Highly relevant with high coherence. Moderate adequacy.
Volume of cases	Pre-existing high volume of cases Increased operative volume	^28,31,37,43,47^	Moderate	Five studies with minor to moderate methodological limitations. Moderate relevance with moderate coherence. Moderate adequacy.
**Organizational**				
Hospital benefit	Increased publicity and reputation benefit Economic benefit Increased competitiveness Delivery of specialized services Research profile for hospital	^28,31,35,41,43,44,47,48^	High	Eight studies with minor to moderate methodological limitations. Highly relevant with high coherence. Moderate adequacy.
Hospital support	Administrative and management support Financial support Facility support Educational support	^25,27,28,30,31,37,38,41,42,43,44,47^	High	Twelve studies with minor to significant methodological limitations. Highly relevant with high coherence. High adequacy.
Interdisciplinary approach	Appropriate team members selected to take part Personal attributes and attitudes of relevant staff Knowledge gained from colleagues in other specialties Culture to allow innovation and change	^28,33,34,42,50^	Moderate	Five studies with minor to moderate methodological limitations. Moderate relevance. High coherence. Moderate adequacy.
Knowledge sharing	Interdepartmental communication Knowledge sharing between participants and other stakeholders (for example, administrators)	^28,38,41,42,43,44,47,48,50^	Moderate	Nine studies with minor to moderate methodological limitations. Moderate relevance. High coherence. Moderate adequacy.
Local clinical outcomes	Reflection of experiences and clinical outcomes, including feedback to relevant stakeholders Clinical audit Accountability for non-compliance	^34,42,47^	Low	Three studies with minor to moderate methodological limitations. Moderate relevance. Moderate coherence. Low adequacy.
Local surgeon champion	Key role to ensure delivery and integration and delivery of adoption of innovation Experience of innovation either during training or from peers	^27,28,30,32,33,34,38,42,47^	High	Nine studies with minor to moderate methodological limitations. Highly relevant with high coherence. High adequacy.
Type of institution	Community or tertiary setting Institutional inertia to change	^25,31,32,37,42,47^	Moderate	Six studies with minor to moderate methodological limitations. Moderate relevance. Moderate coherence. Moderate adequacy.
**Patient-level**				
Patient perspective	Patient choice Patients may wish for innovation if not a candidate for standard treatment Patient perspective that innovation is safer Consumerism Patient satisfaction	^28,29,30,31,33,35,37,39,50^	High	Nine studies with minor to significant methodological limitations. Highly relevant with high coherence. High adequacy.
**Provider-level**				
Level of scientific evidence	Differences in opinion regarding the level of scientific evidence required to support adoption	^35,41,42^	High	Three studies with minor methodological limitations. Highly relevant with high coherence. High adequacy.
Surgeon benefit	Improved ergonomic benefit/working conditions/job satisfaction Increased personal remuneration Increased competitiveness Increased referral rate Research profile for surgeon	^28,31,35,43,48,49,50^	Moderate	Seven studies with minor to moderate methodological limitations. Moderate relevance. Moderate coherence. Moderate adequacy.
Surgeon personal perspectives and experience of innovation	Previous high volume of cases Experience of innovation either during training or from peers Developing or maintaining surgical skills Delivering ‘standard of care’ Desire to be innovative or ‘early-adopter’ Doing ‘what’s best for patient’ Beliefs regarding novelty of adoption (‘newer must be better’)	^25,27,28,30,31,32,35,37,38,43,44,45,46,47,48,50^	High	Sixteen studies with minor to moderate methodological limitations. Highly relevant with high coherence. High adequacy.
**Structural**				
Local social interactions and network	Decision to adopt motivated by informal consensus from local network of physician experiences Local surgeon hierarchy Role of structured decision-making processes	^25,26,28,30,32,35,38,42,44,47,49^	High	Eleven studies with minor to significant methodological limitations. Highly relevant with high coherence. High adequacy.
Manufacturer support	Educational support Resource support	^28,35,41^	Low	Three studies with minor to moderate methodological limitations. Moderate relevance with low coherence. Low adequacy.
Policy support	Hospital-level, governmental-level, regulatory support, and guidelines Processes to facilitate adoption	^28,30,32,34,35,38,41,42,43,44,47^	High	Eleven studies with minor to moderate methodological limitations. Highly relevant with high coherence. High adequacy.
Pressure from external stakeholders	Pressure from external peers, administrators, hospital, insurers, the media, and families of patients Pressure to ‘keep up’	^28,29,32,35,41,46,47^	Moderate	Seven studies with minor to significant methodological limitations. Highly relevant with moderate coherence. Moderate adequacy.
Pressure from internal stakeholders	Pressure from internal peers, administrators, and hospital Attitudes of healthcare providers Identification of local champions to support development	^25,28,29,30,32,33,35,41,46,47,50^	Moderate	Eleven studies with minor to significant methodological limitations. Highly relevant with moderate coherence. Moderate adequacy.

**Table 3:** Themes and sub-themes that are a barrier to adoption of a surgical innovation into clinical practice

Causal factor	Key points	References	Confidence in the evidence	Explanation of confidence finding
**Innovation-based**				
Clinical outcomes	Risk of negative outcomes and associated damage to reputation Concern about overuse/misuse of procedure	^35,37,39,42,48,50^	Moderate	Six studies with minor to significant methodological limitations. Highly relevant with moderate coherence and adequacy.
Cost	Concerns about cost/cost-effectiveness	^26,30,48^	Moderate	Three studies with minor to moderate methodological limitations. Highly relevant with moderate coherence and adequacy.
Education	Lack of training opportunities Long learning curve Difficulty of learning new skills and associated learning curve	^34,40,41,42,43,48,49^	Moderate	Seven studies with minor to moderate methodological limitations. Highly relevant with moderate coherence and adequacy.
Innovation-specific	Technical concerns/challenges with adoption Difficult application of innovation	^30,40,42,48^	Moderate	Four studies with minor to moderate methodological limitations. Highly relevant with moderate coherence and adequacy.
Volume of cases	Low volume of cases and demand for the innovation Conversely, limited time to learn or trial the innovation	^26,30,49^	Low	Three studies with minor to significant methodological limitations. Moderate relevance with low adequacy and coherence.
Workload	Increased workload due to innovation	^39,42,48^	Low	Three studies with minor to significant methodological limitations. Moderate relevance with low adequacy. Low coherence.
**Organizational**				
Geographical isolation	Small community hospitals may lack local social network to support adoption of innovations	^27,35,47^	Moderate	Three studies with minor to moderate methodological limitations. Moderate relevance, coherence, and adequacy.
Hospital organization	Lack of theatre time/facilities/equipment Lack of beds Lack of staff Lack of money Local regulatory processes Lack of management support	^26,27,28,30,31,34,35,37,39,40,42,43,47,48,50^	High	Fifteen studies with minor to significant methodological limitations. Highly relevant with high coherence. High adequacy.
Knowledge sharing	Reduced knowledge sharing between stakeholders and institutions Multiple specialty involvement	^30,35,37^	Moderate	Three studies with minor to moderate methodological limitations. Moderate relevance, coherence, and adequacy.
Staff	Resistance and non-compliance (lack of engagement from stakeholders) Changes in departmental workflow Staff training and education	^42,48,49,50^	Moderate	Four studies with minor methodological limitations. Highly relevant with moderate coherence. Low adequacy.
**Patient-level**				
Local population	Patient preference Patient needs Social characteristics, including age	^27^	Low	One study with moderate methodological limitations. High relevance with low coherence. Low adequacy.
Patient perspective	May choose cheaper standard option over expensive innovation	^26,48,50^	Low	Three studies with moderate methodological limitations. Low relevance with moderate coherence. Low adequacy.
**Provider-level**				
Ethical aspects	Delaying patient access to care to receive innovation potentially causes harm	^30,32,35,48^	Moderate	Four studies.
Medico-legal concerns	Risk of litigation Concerns if limited scientific evidence to support use	^28,35,37,50^	Moderate	Four studies with minor to moderate methodological limitations. Highly relevant with moderate coherence and adequacy.
Senior surgeon view	Limited desire to learn new procedures/skills Limited desire to teach new procedures/skills Decided treatment choice for patients	^26,35,42,47^	High	Four studies with minor to moderate methodological limitations. Highly relevant with moderate coherence. Moderate adequacy.
**Structural**				
Community organization	Lack of support from community services	^27^	Low	One study with moderate methodological limitations. Moderate relevance with low coherence. Low adequacy.
Policy	Adoption barriers from regulatory bodies	^41^	Moderate	One study with minor methodological limitations. Highly relevant with high coherence and adequacy.

#### Innovation-based

Surgeons reported various factors of an innovation that affected their decision to adopt it for use in clinical practice. The most common factor was improved clinical outcomes such as reduced postoperative complication rate, duration of hospital stay, and blood loss, as well as other clinical outcomes leading to reduced time to return to work and overall reduced morbidity and mortality. Some surgeons commented that these could be ‘perceived’ improved outcomes and were not necessarily supported by robust scientific evidence. Surgeons gathered evidence to support their decision-making process from a range of sources (fellow surgeons, local and national conferences, specialty opinion leaders, and peer-reviewed publications). One surgeon stated: ‘*The difference in mortality from an open operation versus [EVAR] is SO huge…the difference between doing open operations and endograft are astronomical. Mortality rates are 3% [EVAR] versus 17 or 18% [open].*’^32^ (where EVAR stands for endovascular aortic aneurysm repair).

An innovation was reported as more likely to be adopted if it improved clinical efficiency such as shorter preoperative, perioperative, and postoperative theatre times, reduced equipment usage, and reduced staff requirements^28,29,31,37,50^. The cost-effectiveness of an innovation was commonly discussed as a factor affecting adoption; particularly if the innovation reduced overall healthcare costs or if it increased reimbursement rates for individual surgeons and institutions^25–28,32,35–37,41,43–45^. Innovations were less likely to be adopted if they were more expensive than the traditional technique. The ease of reimbursement for surgeons and institutions was also cited as a factor positively influencing the diffusion rate.

Successful adoption of an innovation was also related to the complexity of the innovation and the inherent compatibility of the innovation with existing clinical practice. Innovations that required minimal adjustment to existing clinical practice and pathways were more likely to be adopted. Innovations were more likely to be adopted if there was a pre-existing high volume of cases or if surgeons believed that the innovation would lead to increased number of operations.

A major component regarding the ease of adoption is related to the education and training of the individual surgeons. Innovations that were challenging to perform from a technical skill perspective with a long learning curve were less likely to be adopted. One participant noted that innovations often required new knowledge and intensive training: ‘*But at the time we introduced that, when the treatment was new, each component had to be learned first […]*’^41^.

This could be mitigated to some extent by use of formal surgical training pathways, local surgeon champions, simulation and other preparatory resources, and credentialing to demonstrate competence to perform a procedure. One surgeon in their department described their initial experience with the procedure: ‘*We practiced just watching what they were doing, then we did it under x-ray control…then we had a couple of experts come and we did it with them on two patients the next day.*’^32^.

There were some concerns raised regarding the potential risk of negative clinical outcomes and the associated damage to reputation. Other concerns included the potential of overuse or misuse of a procedure before scientific evidence could be demonstrated.

Innovations in surgery can often transcend inter-specialty boundaries (for example, EVAR for an abdominal aortic aneurysm crosses interventional radiology and vascular surgery). Each specialty has unique knowledge and skill sets; therefore, the interplay and linkage between the specialties involved was an important consideration regarding the adoption of an innovation. Management and negotiation of specialty boundaries through effective interdisciplinary communication was important to the adoption of an innovation. Determining which specialty ‘owns’ the innovation can be challenging, particularly when multiple specialties already perform the procedure. Inter-specialty teamwork often influenced the adoption decision and slowed down or interrupted the implementation process: ‘*These are the nuts and bolts…Because, often the teamwork of cardiologists and cardiac surgeons is not given…This is a major problem because interventional cardiology has expanded into many areas of heart surgery…*’^41^.

Benefits of an interdisciplinary approach include the important selective expertise of different specialties in managing patients: ‘*It’s amazing, the endoscopic techniques we use are similar to those used in pulmonary. Why would a urologist be hanging out with a pulmonologist? You’d be surprised, they’re using a lot of similar things, so there’s a lot of cross-over. We don’t necessarily need always to look within our specialty, but also to look outside our specialty for ideas.*’^49^.

Barriers to the optimal development of interdisciplinary teams include logistic issues such as institutional insight, overcoming inertia, and traditional professional boundaries. Non-logistic barriers include remuneration structures and institutional resources to accommodate interdisciplinary teams. Adoption strategies that encouraged staff to ‘own’ the innovation and take responsibility for it were helpful.

#### Organizational

The role of the healthcare provider institution is integral to the decision to adopt a novel surgical innovation. Multiple surgeons mentioned the need for institutional oversight and support in terms of finance, administration, management, education, and logistics. Institutions that were unable to support these factors encountered significant barriers to adoption. Local regulatory processes presented administrative burdens to innovation.

The type of institution (academic *versus* community), geographical location, and the associated levels of support (both in-hospital and community) that could be provided affected the decision to adopt or not adopt certain innovations. Surgeons mentioned the benefits to individual institutions from adopting an innovation, including increased publicity and reputation, increased competitiveness, increased research profile, potential economic benefit, and the ability to deliver specialized services. The institution’s strategic focus needs to be taken into account, particularly when institutions focus on one area (for example, oncology) and are ‘pro-innovation’ in order to provide the latest standard of care. One surgeon commented: ‘*Cancer surgery is considered to be one of our strategic priorities. So because laparoscopic surgery is involved with cancer surgery, it just fits in.*’^44^.

Conversely, innovations introduced into rarer surgeries with fewer patients and fewer referrals hindered sustained adoption due to low use of the new technique: ‘*I still wasn’t as busy with [novel procedure] as I would want to be, and so it might be several weeks or sometimes even a month or two between cases. So, the ability to rapidly turn around on surgical learning was not as available.*’^48^.

The role of a local surgeon champion or leader was consistently mentioned by surgeons as driving the adoption of an innovation. This refers to surgeons within a department who have had a significant influence on the novel innovation, including experience in its implementation and significant experience of using an innovation. Therefore, these champions could provide motivation and invaluable support to other surgeons. In one study, a surgeon was hired specifically to perform endovascular repairs and was instrumental to its early use: ‘*The thing that really kick started it is when we hired a partner who had been trained to do this in the US. So he had done a lot of these cases, and so we then went fairly rapidly and ramped up the program.*’^32^.

There were several communication-related sub-themes that were central to adoption of surgical innovation. Knowledge sharing was key between all the participants and stakeholders, including hospital administration and management, to identify and solve implementation issues. This could take the form of regular multidisciplinary meetings, promoting a positive culture of communication. Multidisciplinary meetings also provided valuable forums to reflect on experiences and clinical outcomes of an innovation. Clinical audit of outcomes was important, as was accountability for non-compliance with an innovation. The overall stability of staff and their culture to allow innovation and change all facilitated the adoption of an innovation. Lack of engagement from relevant stakeholders and resistance to change were identified as a barrier to adoption of an innovation.

#### Patient-level

A number of themes related to individual surgeons or patients emerged from the data. The patient perspective was integral, as patients often expressed a wish for a specific treatment that they had heard about. Several surgeons reported that patients believed ‘innovations were safer’ and there was an element of consumerism regarding patient choice of treatment. As one patient put it: ‘*the word “robot”, of course, sounds **magical** [strong verbal emphasis]. [It] suggests that things can’t go wrong anymore*’^28^.

Certain patients elected for an innovative treatment if they were not candidates for standard treatment. This sometimes led to ethical issues, as some surgeons felt that delays in accessing care, in order to receive the innovative treatment, could contribute to patient harm through increased morbidity and mortality. In healthcare systems where patients were responsible for paying for their healthcare, many patients chose the cheaper standard option over the expensive innovative technology. The social and geographical characteristics of the local patient population also directly affected the adoption of an innovation. One study reported that the adoption of their innovation followed the ‘medical-individualistic’ perspective, which focuses on hospitals adopting new technologies based on the clinical needs of their patient population and the benefit of the intervention for the patient over economic considerations^32,38^.

#### Provider-level

The personal attitudes, views, experiences, motivators, and characteristics of individual surgeons were also prominently mentioned. Surgeons with previous experience and a high volume of cases using an innovation were more likely to adopt a procedure into their clinical practice. Experience could come from training opportunities or local peers. Surgeons’ personal beliefs regarding innovation also affected the decision to adopt an innovation; some surgeons had a desire to be innovative or an ‘early-adopter’, whilst others adopted an innovation to continuously develop or maintain their technical surgical skills^49^. For some surgeons, their personal belief was patient-focused and their decision to adopt an innovation was related to ‘doing what’s best for the patient’^31,35^. Some surgeons held beliefs regarding the novelty aspect of an innovation and that ‘newer must be better’^28^. Some surgeons commented on the personal benefits to them from adopting an innovation into practice, including practical considerations such as improved working conditions, job satisfaction, and lifestyle benefits, and economic considerations such as increased personal remuneration, increased competitiveness for cases, and increased referral rates. Surgeons also considered the increased research profile they could gain from adopting a surgical innovation. Innovations that increased workloads faced resistance to adoption.

There was no clear consensus regarding the strength of evidence required to support or oppose an innovation’s effectiveness. Decisions to adopt a new technology were often made with limited, if any, scientific evidence of efficacy. One surgeon highlighted the urgency to adopt the technology before RCT evidence: ‘*It just is something that made intuitive sense and you could sit on the sidelines and wait for randomized data and clinical trials, but you’d be waiting for a really long time and I’m in the trenches operating on people now and looking at them now.*’^35^.

Many surgeons preferred RCTs, but others were happy with data from other sources such as observational data or personal intuition and experience. Some surgeons relied on the community standard of care before adopting an innovation into their own practice. There are also challenges of generalizing evidence about interventions from academic to community settings.

##### Ethical issues

Several studies reported on ethical considerations affecting the decision to adopt or reject a novel innovation. These concerns related to delays or issues accessing care in order to receive the innovative treatment. One study explained: ‘*At times, referring the patient for EVAR assessment across hospital boundaries might cause delays in decision making, challenging the hand-over of patient responsibility. This in turn could be critical in the case of an aneurysm rupture.*’^30^.

Critical appraisal and analysis of the evidence of benefit from the scientific literature differed between individual surgeons. A radiation oncologist who was medical director of oncology for a large group represented a more conservative approach and said: ‘*I’ve probably slowed down some of the desire to do things more quickly and push more towards the trial scenario…and look that direction first.*’^35^.

These findings highlight that adoption of a surgical innovation transcends both clinical care and research. It is not always clear when formal ethical committee oversight is required (for example, should a minor modification to an existing procedure require ethical oversight or robust health technology assessment, or neither). Part of this confusion may result from the lack of uniformity in surgeons’ views regarding the definition of ‘surgical innovation’^51^. A systematic review investigating the ethical issues associated with surgical innovation highlighted four major themes: oversight of the surgical innovation process; informed consent; learning curve of the individual surgeons; and issues surrounding innovative procedures in vulnerable patients^52^.

##### Medico-legal concerns

A number of surgeons reported concerns regarding litigation and medico-legal issues resulting from the adoption of surgical innovations. There were concerns regarding the implication of using an innovation that had limited scientific evidence to support its use.

#### Structural

Surgeons reported that local social interactions within their network directly affected the adoption of an innovation. Many surgeons reported that the continued decision to adopt an innovation was often motivated by informal consensus and experiences from surgeons and other interdisciplinary peers within their individual social network. This was not necessarily restricted to their own institutions. Indeed, many surgeons described that the pressure from internal and external stakeholders was directly relevant to their decision to adopt an innovation. Internal stakeholders included groups such as patients, fellow surgeons, administrators, hospital managers, etc. Their attitudes towards innovation and novel technology affected the pressure they exerted. Pressure also came from external stakeholders such as insurers, device manufacturers, policymakers, relevant national societies, families of patients, and the media. Surgeons described a pressure to ‘keep up’ with current practice, even in the absence of scientific evidence. Several surgeons mentioned surgical hierarchy and the associated impact of the opinion of a senior surgeon within the department. If a senior surgeon did not utilize an innovation themselves then it was unlikely that junior surgeons or trainees would use the innovation themselves. Senior surgeons had their own individual motivations with some surgeons discussing their own limited desire to learn or teach new procedures and skills. One senior surgeon noted: ‘*I think [the reason why team C was not selling laparoscopy very much was] just a lack of interest because Dr. X (senior surgeon), who was trained in it, perhaps looking back, maybe we should have trained somebody else. He was trained in it, yet he hardly performed any…I think he feels he’s retired, I don’t think he wants to go through all the trouble…, so I think he thinks there is no time for him to do it.*’^26^.

The role of formal policy and guidelines featured in many surgeons’ desires to adopt a surgical innovation. Policy support and guidelines could come from many sources, including hospitals, local and national government, funding bodies, and regulatory bodies. One study reported that the lack of governmental support for an adoption led to a decision to cut the funding for it^32^. The importance of national device regulation influenced diffusion rate, as one study noted that: ‘*The approval system for medical devices in Europe was…described as “innovation friendly” in comparison to the US medical device regulation.*’^41^.

There were mixed views on the role of a formal process to facilitate adoption of an innovation. Some surgeons thought that the adoption of an innovation would have been facilitated by a formal process to ensure a fair decision-making process^32^. One study reported that the introduction of an innovation in a new unit: ‘*without entrenched rules or leaders, helped to allow it to grow and spread*’^38^.

Surgeons also considered the role of device manufacturers in providing educational and resource support to facilitate adoption of their products.

## Discussion

This systematic review has synthesized the evidence related to surgeons’ views of factors affecting the adoption of a novel surgical innovation into clinical practice. The themes identified have been classified using the framework of Chaudoir *et al*.^9^.

This study contributes to the body of evidence regarding the diffusion and adoption of surgical innovations by exploring surgeons’ perspectives. It shows that there are multiple factors affecting a surgeon’s decision to use a novel surgical innovation and the complex interplay between these factors will lead to sustainability of the innovation or its subsequent demise. Key themes identified with confidence that facilitate adoption include improved clinical outcomes from the innovation, cost-effectiveness, scientific evidence, surgeon experience, views and beliefs regarding the innovation, the local social network, and the role of surgeon champions, as well as pressure and support from internal and external stakeholders. Barriers to the adoption of a surgical innovation such as the views of senior surgeons and the lack of resources or organizational support were reported in the majority of studies.

Given the importance of clinical outcomes regarding a surgeon’s decision to adopt a novel technology, it is interesting to note the lack of consensus amongst surgeons regarding the strength of evidence required to confidently make this decision. A qualitative study investigating the diffusion of service innovations noted that ‘The relative advantage of healthcare innovations, and even the evidence of benefit, is not a judgement rooted in pure rationalistic reasoning, but rather is subject to debate and negotiation’^53^. Studies investigating the impact of scientific information on the diffusion of healthcare innovations have found that robust scientific evidence does not necessarily lead to innovation adoption in a linear or direct way.

There is a need to develop the insight and understanding of the strength of evidence required to support the widespread adoption of a novel surgical technology. Knowledge of the strength of evidence required for stakeholder support will inform the design, methodology, scope, and objectives of future clinical trials (for example, efficacy *versus* effectiveness trials)^54^. The IDEAL framework describes the stages of evidence generation for novel surgical innovations; however, this framework does not currently integrate well with medical regulatory bodies, which offer different guidance and evaluation of an interventional procedure depending on its stage of development^1,7^. It is crucial to understand the complex interplay between the surgeon’s perspective of the level of evidence, the regulator’s perspective of the level of evidence, and the clinical circumstances needed to move between stage 3 and stage 4 of the IDEAL framework. Adoption of surgical innovations may have significant resource implications for individual institutions and the entire healthcare system; therefore, the decision-making processes of surgeons should be overseen^55^. Understanding what scientific data should be available for a new surgical innovation before it can be used widely in the National Health Service, in the views of multiple different stakeholders, will help inform the design, methodology, scope, and objectives of future clinical trials (for example, efficacy *versus* effectiveness trials)^54^. It is encouraging that the use of the IDEAL framework in surgical innovation is accelerating^56^.

The themes and sub-themes identified in this review are consistent with those identified by other studies exploring innovation in healthcare^9,10,23,57,58^. This review provides deeper insights into the views and perspectives of the individual surgeons who are faced with the decision of whether to use a novel surgical innovation in a clinical scenario. This review contributes to the literature regarding evidence-based surgical innovation.

This review has several strengths. A comprehensive search using standard methodology was conducted to identify studies directly relevant to the research question. Two independent authors were involved in extraction, analysis, and interpretation to ensure that the synthesis incorporated the breadth and depth of experiences reported in the studies. This review synthesized data from 1112 participants (of which 694 were surgeons) across different healthcare systems. A protocol paper was published before this study^11^. Although the protocol paper included searches of grey literature in the Methods section, these data were not searched systematically and not used in the final data analysis or Results section.

There are, however, some limitations. The range of surgical innovations investigated by the included studies was small and therefore not necessarily generalizable to other surgical innovations. Types of innovation included innovations involving endoscopic techniques (6), endovascular innovations (3), robotic innovations (2), organizational innovations (7), general surgery innovations (4), and innovations involving other specialties (4). Similarly, the majority of the studies were conducted in high-income Western countries, with only one study conducted in a low-income country, which potentially restricts the transferability of the review findings and the evidence base. In total, 14 of the studies included views from other healthcare staff in addition to surgeons. Statements from interviews were often vague, with authors stating improved health outcomes or lower overall healthcare costs, without giving concrete justification to support these statements. Furthermore, a broad range of keywords are often used in this area of research and studies were excluded if they did not include surgeons’ opinions. Process evaluations for new technologies may further assist the thought process behind decisions to adopt new innovations^59–62^.

Surgeons’ views and perspectives about factors that affect their decision to adopt a novel surgical innovation focus predominantly on the innovation itself, particularly the potential improvement of clinical outcomes. However, there are many more factors that affect this decision at patient, provider, organizational, and structural levels. An increased understanding between patients, surgeons, researchers, regulators, commissioners, and other relevant stakeholders of these factors should play an integral role in future strategies and research into the diffusion and adoption of surgical innovations.

This thematic synthesis of qualitative studies on surgeons’ perspectives shows that there are multiple complex factors that dynamically interact, affecting the adoption of a novel surgical innovation into clinical practice. These can be categorized according to innovation-based, provider-level, patient-level, organizational, and structural causal factors. Clarification is still needed as to the strength of clinical evidence needed to support the adoption of new innovations. These insights will be valuable in understanding how best to implement surgical innovations in an effective, but also safe, manner.

## Supplementary Material

zrad141_Supplementary_Data

## Data Availability

The data that support the findings of this study are available on request from the corresponding author (A.J.M.).
