# Supplementary material for: Surgeon views regarding the adoption of a novel surgical innovation into clinical practice: systematic review
Source: BJS Open. 2024 Jan 24;8(1):zrad141. doi: 10.1093/bjsopen/zrad141 (PMC10807848; doi:10.1093/bjsopen/zrad141)
Supplement: zrad141_Supplementary_Data [file zrad141_supplementary_data.docx]

**SURGEON VIEWS REGARDING THE ADOPTION OF A NOVEL SURGICAL INNOVATION INTO CLINICAL PRACTICE: SYSTEMATIC REVIEW OF QUALITATIVE RESEARCH**

NN Konda^1,2^, TL Lewis^3^, HN Furness^4^, GW Miller^5^, AJ Metcalfe^1,2^, DR Ellard^1, 2^

1. Mr Nagarjun N Konda

Core Surgical Trainee

[Nagarjun.konda1@nhs.net](mailto:Nagarjun.konda1@nhs.net)

ORCID: <https://orcid.org/0000-0002-8801-1278>

1. Mr Thomas L Lewis

ST7 Trauma and Orthopaedic Surgery Specialty Trainee

[Email: t.lewis2@nhs.net](mailto:Email:%20t.lewis2@nhs.net)

ORCID: [orcid.org/0000-0002-4167-7427](https://gbr01.safelinks.protection.outlook.com/?url=http%3A%2F%2Forcid.org%2F0000-0002-4167-7427&data=05%7C01%7Cnagarjun.konda1%40nhs.net%7C2fa773f7f8714acef81108db466455d1%7C37c354b285b047f5b22207b48d774ee3%7C0%7C0%7C638181168966050786%7CUnknown%7CTWFpbGZsb3d8eyJWIjoiMC4wLjAwMDAiLCJQIjoiV2luMzIiLCJBTiI6Ik1haWwiLCJXVCI6Mn0%3D%7C3000%7C%7C%7C&sdata=5jzGluCH5ZhZAI%2FxMo6qqQnRQ8Kc14kEq%2BVKlKE8d9w%3D&reserved=0)

1. Dr Hugh N Furness

Research Physician

[hughfurness@hotmail.com](mailto:hughfurness@hotmail.com)

1. Dr George W Miller

Core Surgical Trainee

george.w.miller1@googlemail.com

1. Professor Andrew J Metcalfe (corresponding author)

Professor of Trauma and Orthopaedics

a.metcalfe@warwick.ac.uk

1. Professor David R Ellard

Professor of clinical trial methodology

[D.R.Ellard@warwick.ac.uk](mailto:D.R.Ellard@warwick.ac.uk)

ORCID: <https://orcid.org/0000-0002-2992-048X>

^1^Warwick Clinical Trials Unit, Warwick Medical School, The University of Warwick, Coventry, CV4 7AL, UK

^2^Dept. of Trauma and Orthopaedic surgery, University Hospitals Coventry & Warwickshire, Clifford Bridge Road, Coventry, CV2 2DX, UK

^3^King’s College Hospital NHS Foundation Trust, London, SE5 9RS

^4^Imperial College London, Exhibition Rd, London SW7 2BX

^5^Bart’s and The London NHS Foundation Trust, The Royal Hospital, Whitechapel Rd, London E1 1BB

**Corresponding author:**  Professor Andrew J Metcalfe

**Supplementary Materials - Index**

| **Supplementary Methods** |  |
| --- | --- |
| Table S1: Search Strategy | *page 4-5* |
| Figure S1: Risk of bias analysis | *page 6-7* |
|  |  |

**Table S1: Search strategy**

| **MEDLINE** | **EMBASE** | **COCHRANE LIBRARY OF SYSTEMATIC REVIEWS** | **SCOPUS** | **WEB OF SCIENCE** |
| --- | --- | --- | --- | --- |
| 1. adoption.mp. or exp Adoption/  2. diffusion.mp. or exp Diffusion/ or exp "Diffusion of Innovation"/  3. dissemination.mp. or exp Information Dissemination/  4. introduction.mp. | 1. adoption.mp. or exp adoption/  2. exp diffusion/ or diffusion.mp.  3. dissemination.mp. or exp information dissemination/  4. introduction.mp. | 1. adoption  2. diffusion  3. dissemination  4. introduction | TITLE-ABS-KEY((adoption OR diffusion or dissemination or introduction) AND (Novel or new or innovat*) AND (surg* w/5 (procedure or operation or instrument or technique or device or implant or technolog* or intervention)) and (view* or perspective or qualitative or attitude or preference or choice)) | (TS=((adoption OR diffusion or dissemination or introduction) AND (Novel or new or innovat*) AND (surg* NEAR/5 (procedure or operation or instrument or technique or device or implant or technolog* or intervention)) and (view* or perspective or qualitative or attitude or preference or choice))) AND LANGUAGE: (English) |
| 5. 1 or 2 or 3 or 4 | 5. 1 or 2 or 3 or 4 | 5. #1 or #2 or #3 or #4 |  |  |
| 6. novel.mp.  7. new.mp.  8. exp Organizational Innovation/ or exp "Diffusion of Innovation"/ or innovat*.mp. | 6. novel.mp.  7. new.mp.  8. innovat*.mp. | 6. novel  7. new  8. innovat* |  |  |
| 9. 6 or 7 or 8 | 9. 6 or 7 or 8 | 9. #6 or #7 or #8 |  |  |
| 10. surg*.mp. or exp General Surgery/  11. procedure.mp.  12. operation.mp.  13. exp Surgical Instruments/ or instrument.mp.  14. technique.mp.  15. device.mp. or exp "Equipment and Supplies"/  16. implant.mp. or exp Bone-Implant Interface/  17. exp Technology Transfer/ or exp Technology Assessment, Biomedical/ or exp Educational Technology/ or exp Biomedical Technology/ or technolog*.mp. or exp Technology, High-Cost/ or exp Technology/ or exp Technology, Pharmaceutical/ or exp Wireless Technology/ or exp "National Center for Health Care Technology (U.S.)"/ or exp "United States Office of Technology Assessment"/ or exp Technology, Radiologic/ or exp Remote Sensing Technology/  18. intervention.mp. | 10. surg*.mp. or exp surgery/  11. procedure.mp. or exp procedures/  12. operation.mp.  13. instrument.mp.  14. technique.mp.  15. device.mp. or exp devices/  16. exp implant/ or implant.mp.  17. exp technology/ or technolog*.mp.  18. intervention.mp. | 10. surg*  11. procedure  12. operation  13. instrument  14. technique  15. device  16. implant  17. technolog*  18. intervention |  |  |
| 19. (surg*.mp. or exp General Surgery/) adj5 ((procedure or operation).mp. or (exp Surgical Instruments/ or instrument.mp.) or technique.mp. or (device.mp. or exp "Equipment and Supplies"/) or (implant.mp. or exp Bone-Implant Interface/) or (exp Technology Transfer/ or exp Technology Assessment, Biomedical/ or exp Educational Technology/ or exp Biomedical Technology/ or technolog*.mp. or exp Technology, High-Cost/ or exp Technology/ or exp Technology, Pharmaceutical/ or exp Wireless Technology/ or exp "National Center for Health Care Technology (U.S.)"/ or exp "United States Office of Technology Assessment"/ or exp Technology, Radiologic/ or exp Remote Sensing Technology/) or intervention.mp.) | 19. (surg*.mp. or exp surgery/) adj5 (procedure.mp. or exp procedures/ or operation.mp. or instrument.mp. or technique.mp. or (device.mp. or exp devices/) or (exp implant/ or implant.mp.) or (exp technology/ or technolog*.mp.) or intervention.mp.) | 19. #10 near/5 (#11 or #12 or #13 or #14 or #15 or #16 or #17 or #18) |  |  |
| 20. view*.mp.  21. perspective.mp.  22. exp Qualitative Research/ or qualitative.mp.  23. opinion.mp. or exp "Attitude of Health Personnel"/  24. exp "Attitude of Health Personnel"/ or exp Attitude/ or attitude.mp.  25. preference.mp.  26. exp Choice Behavior/ or choice.mp. | 20. view*.mp.  21. perspective.mp.  22. qualitative.mp. or exp qualitative analysis/ or exp qualitative research/  23. opinion.mp.  24. exp health personnel attitude/ or exp physician attitude/ or attitude.mp. or exp attitude/ or exp attitude to change/  25. preference.mp.  26. choice.mp. | 20. view*  21. perspective  22. qualitative  23.opinion  24.attitude  25. choice  26. preference |  |  |
| 27. 20 or 21 or 22 or 23 or 24 or 25 or 26 | 27. 20 or 21 or 22 or 23 or 24 or 25 or 26 | 27. #20 or #21 or #22 or #23 or #24 or #25 or #26 |  |  |
| 28. 5 and 9 and 19 and 27 | 28. 5 and 9 and 19 and 27 | 28. #5 and #9 and #19 and #27 |  |  |
| 29. limit 28 to (english language and humans) | 29. limit 28 to (human and english and (article or books or chapter or editorial or letter or reports or "review" or short survey)) |  |  |  |
| **718** | **3034** | **361** | **871** | **369** |
| limit 28 to (english language and humans and 2017-current) | limit 28 to (human and english and (article or books or chapter or editorial or letter or reports or "review" or short survey)) and 2017 to current | Since September 2017 | 2017-2020 | 2017-2020 |
| **216** | **1182** | **107** | **302** | **155** |
| limit 28 to (english language and humans and yr="2018 -Current") | limit 28 to (human and english language and yr="2018 -Current" and (article or books or chapter or editorial or letter or "review" or short survey)) |  | 2018-2020 | 2018-2020 |
| **168** | **929** | **As above** | **212** | **114** |
| Limit 28 to dt=20170901-20171231 | limit 28 to dd=20170901-20171231 |  |  |  |
| **23** | **18** | **As above** | **Not possible** | **Not possible** |
|  |  |  | 2020-Present | 2020-2020 |
| **97** | **9** |  | **101** | **62** |

**Figure S1: Risk of bias analysis**

**Confidence in review findings**

The GRADE-CERQual framework has been utilised to asses confidence in the findings found in Table 2 and 3 within this review(45). The majority of the findings in Table 2 were rated moderate to high confidence. The majority of findings in Table 3 were rated moderate to low confidence. This was predominantly due to studies preferentially reporting positive themes that facilitated the adoption of surgical innovation rather than the barriers to adoption.

*Methodological limitations:*

Each study underwent quality assessment according to a CASP checklist(42,44). Studies were found to have a range of minor to significant methodological limitations. Caution was taken when interpreting the results of studies with significant methodological limitations.

*Coherence:*

There is considerable coherence in the data presented in the studies. Common subthemes were easily identified from the studies. These subthemes are also recognised by other studies investigating the diffusion of innovation in healthcare leading to increased confidence in these findings(3,10–14).

*Adequacy of data:*

The findings of this review are drawn from a number of studies that contain ‘rich data’. A small number of the studies included did not provide “rich data” however they did still echo the findings of the “rich data” studies. It is unlikely that additional data from these studies would have significantly changed our findings given the comparative detail of information provided in other studies.
